# Southern ocean carbon and heat impact on climate

**DOI:** 10.1098/rsta.2022.0056

**Published:** 2023-06-26

**Authors:** The SO-CHIC consortium, J. B. Sallée, E. P. Abrahamsen, C. Allaigre, M. Auger, H. Ayres, R. Badhe, J. Boutin, J. A. Brearley, C. de Lavergne, A. M. M. ten Doeschate, E. S. Droste, M. D. du Plessis, D. Ferreira, I. S. Giddy, B. Gülk, N. Gruber, M. Hague, M. Hoppema, S. A. Josey, T. Kanzow, M. Kimmritz, M. R. Lindeman, P. J. Llanillo, N. S. Lucas, G. Madec, D. P. Marshall, A. J. S. Meijers, M. P. Meredith, M. Mohrmann, P. M. S. Monteiro, C. Mosneron Dupin, K. Naeck, A. Narayanan, A. C. Naveira Garabato, S-A. Nicholson, A. Novellino, M. Ödalen, S. Østerhus, W. Park, R. D. Patmore, E. Piedagnel, F. Roquet, H. S. Rosenthal, T. Roy, R. Saurabh, Y. Silvy, T. Spira, N. Steiger, A. F. Styles, S. Swart, L. Vogt, B. Ward, S. Zhou

**Affiliations:** ^1^ Laboratoire d’Océanographie et du Climat Expérimentations et Approches Numériques (LOCEAN), Sorbonne Université, CNRS/IRD/MNHN, Paris, France; ^2^ British Antarctic Survey, Cambridge, UK; ^3^ European Polar Board, Den Haag, The Netherlands; ^4^ Department of Marine Sciences, University of Gothenburg, Gothenburg, Sweden; ^5^ University of Reading, Reading, UK; ^6^ ETH Zürich, Zürich, Switzerland; ^7^ Alfred Wegener Institute Helmholtz Centre for Polar and Marine Research, Bremerhaven, Germany; ^8^ National Oceanography Centre, Southampton, UK; ^9^ University of Oxford, Oxford, UK; ^10^ Southern Ocean Carbon-Climate Observatory (SOCCO), CSIR, Cape Town, South Africa; ^11^ University of Southampton, Southampton, UK; ^12^ ETT, Genoa, Italy; ^13^ GEOMAR Helmholtz Centre for Ocean Research Kiel, Kiel, Germany; ^14^ Norwegian Research Centre (NORCE), Bergen, Norway; ^15^ ECOCEANA, Paris, France; ^16^ Department of Oceanography, University of Cape Town, Rondebosch, South Africa; ^17^ AirSea Laboratory and Ryan Institute, School of Natural Sciences, University of Galway, Galway, Ireland; ^18^ Department of Oceanography, Dalhousie University, Halifax, Canada; ^19^ IBS Center for Climate Physics and Department of Climate System, Pusan National University, Busan, Republic of Korea

**Keywords:** Southern Ocean, climate, ocean heat storage, ocean carbon storage

## Abstract

The Southern Ocean greatly contributes to the regulation of the global climate by controlling important heat and carbon exchanges between the atmosphere and the ocean. Rates of climate change on decadal timescales are therefore impacted by oceanic processes taking place in the Southern Ocean, yet too little is known about these processes. Limitations come both from the lack of observations in this extreme environment and its inherent sensitivity to intermittent processes at scales that are not well captured in current Earth system models. The Southern Ocean Carbon and Heat Impact on Climate programme was launched to address this knowledge gap, with the overall objective to understand and quantify variability of heat and carbon budgets in the Southern Ocean through an investigation of the key physical processes controlling exchanges between the atmosphere, ocean and sea ice using a combination of observational and modelling approaches. Here, we provide a brief overview of the programme, as well as a summary of some of the scientific progress achieved during its first half. Advances range from new evidence of the importance of specific processes in Southern Ocean ventilation rate (e.g. storm-induced turbulence, sea–ice meltwater fronts, wind-induced gyre circulation, dense shelf water formation and abyssal mixing) to refined descriptions of the physical changes currently ongoing in the Southern Ocean and of their link with global climate.

This article is part of a discussion meeting issue ‘Heat and carbon uptake in the Southern Ocean: the state of the art and future priorities’.

## 1. Introduction

In the past 60 years, the ocean has taken up and stored 23±5% of anthropogenic carbon emissions, and since the 1970s, more than 90% of the heat that has accumulated in the Earth system [1,2]. This tandem of heat and ${\mathrm{CO}}_{2}$ uptake by the ocean has played a major role in setting the rate of global warming, and therefore the ocean processes controlling this ocean carbon–heat nexus are central to our understanding of global climate change under increasing greenhouse gas emissions or after emission cessation (e.g. [1,3–5]).

The key role of the ocean in shaping the response of global surface air temperature to greenhouse gas emissions can be expressed through a simple linear energy budget equation that explains global warming (ΔT) as the result of an effective radiative forcing at the top of the atmosphere (ΔF; modulated, in part, by ocean carbon uptake) minus the energy that is absorbed by the global ocean (ΔN; directly the result of ocean heat uptake), with amplification or dampening by physical climate feedbacks (α; set, in part, by ocean surface temperature through the so-called ‘pattern effect’ (e.g. [6–8]))

1.1 $$\Delta T=\frac{\Delta F-\Delta N}{\alpha }.$$

Across the global ocean, the Southern Ocean, defined as the ocean south of $30^{\circ }\;S$, has a particularly large role in affecting each of the three terms of equation (1.1) that control global warming. The Southern Ocean is responsible for about 70±30% of the excess heat absorbed by the world’s ocean each year (e.g. [9–11]) and about half of the uptake of anthropogenic carbon [12–14]. It has also been recognized to have a profound influence on decadal-scale variability of atmospheric ${\mathrm{CO}}_{2}$ concentrations, which are controlled, at first order, by variability in the Southern Ocean circulation [15–18]. The Southern Ocean is a large anthropogenic carbon sink and a key region of uncertainty in computation of the global ocean carbon uptake, that urgently requires significant advancements in both modelling and observation [12,19]. Finally, the Southern Ocean has a large impact on physical climate feedback, such as low cloud feedback, that depends on the spatial patterns of sea surface temperature (e.g. [20,21]).

Despite the recognition of its large impact on climate, the Southern Ocean remains poorly observed and understood, and insufficiently well represented in climate models. While enhanced heat and carbon uptake by the Southern Ocean are ubiquitous features of climate change simulations [10,22], the magnitude and pattern of heat and carbon uptake vary enormously between different models. For instance, in the model suite of phase 5 of the Coupled Model Intercomparison Project (CMIP5), the Southern Ocean stands out as the region where models differ the most in their representation of anthropogenic ${\mathrm{CO}}_{2}$ and heat uptake [1,2,10]. In particular, the inter-model standard deviation of the heat uptake is as high as ±40% of the multi-model mean over the historical period [10].

The central element that inhibits our understanding and ability to predict decadal-to-centennial variability of ocean heat and carbon uptake and storage is our lack of understanding of the rate at which waters are ‘ventilated’, i.e. the rate at which atmospheric signal and energy reach the deep sea, and conversely, the rate at which the deep-sea climate signal can reach the surface ocean to connect with the atmosphere. The Southern Ocean provides a preferential pathway for ventilation, with more than 60% of the world’s ocean waters having their last contact with the atmosphere in this region [23]. Carbon and heat ventilation involves two main steps: (i) fluxes through the ocean surface [24] and (ii) transfer from the surface boundary layer to the deeper ocean, where such tracers are isolated from the atmosphere. The transfer from the surface boundary layer of the Southern Ocean to deeper waters can take place either in the open seas through mixing and subduction [25,26], or along Antarctic margins through the formation and export of dense bottom waters [27]. Both ventilation pathways are sensitive to a range of complex dynamical processes and to perturbations of surface buoyancy fluxes and winds (e.g. [28–30]).

In addition to these large-scale processes, the Southern Ocean climate system contains components with highly nonlinear behaviour that may lead to abrupt, dramatic changes in anthropogenic heat and carbon release back to the atmosphere—and which may represent an ‘Achilles heel’ of the system. Heat stored in the Southern Ocean may be released whenever the open-ocean Weddell Polynya (i.e. a large-scale ice-free area within closed sea–ice cover) opens and deep convection brings warm deep water into contact with the atmosphere [31]. Since it was first observed in the mid-1970s [32], such a large winter-persistent polynya has not reappeared. It has been hypothesized that a shift towards warmer climates could prevent it from opening for centuries due to the strengthening of the halocline [33]. However, a short-lived polynya formed over Maud Rise during the austral winters of both 2016 and 2017 [34] and, more recently, another polynya appeared in late austral spring (November) 2021. It is an urgent priority to instrument the area to observe in detail how the ocean stratification has been affected, how water mass transformation in the region is impacted and what is the likelihood of the re-occurrence of a large polynya [35].

To contribute to reducing uncertainties in climate change predictions and to allow governments and policymakers to take informed decisions related to climate change, the Southern Ocean Carbon and Heat Impact on Climate programme was launched (SO-CHIC; see the electronic supplementary material). In this paper, we present some of the advances in understanding made in the first half of the programme.

## 2. The SO-CHIC programme

The overall objective of SO-CHIC is to understand and quantify the variability of heat and carbon budgets in the Southern Ocean. To address this central objective, SO-CHIC aims at meeting five specific objectives:

O1. To initiate sustained monitoring of budgets of heat and carbon in the Southern Ocean, by quantifying their fluxes at the air–sea–ice interface and estimating interannual variability of heat and carbon storage in the Southern Ocean.
O2. To improve understanding of the spatial distribution and variability of heat and carbon exchanges between the atmosphere and the deep ocean, focusing on the dynamics of the ocean mixed-layer and its relation to sea ice distribution, and on assessing what has caused the opening of an open-ocean polynya above Maud Rise in 2016 and 2017, more than 40 years after the previous major polynya event.
O3. To improve understanding of the formation and export of bottom waters in the Bottom Boundary Layer, which ventilate the world’s abyssal ocean, and propose new strategies to represent such key processes, which is one major failure of current state-of-the-art climate models.
O4. To identify critical sensitivities in the Southern Ocean climate system that must be correctly represented in models in order to significantly reduce uncertainties in future projections of oceanic heat and carbon content.
O5. To enable free and open access to all data and maximize impact on climate reports (IPCC), climate services and climate-model groups.

To reach these goals, SO-CHIC uses a combination of observations and modelling efforts (electronic supplementary material). The concept is not solely to try to observe some specific processes, but to build a strategic approach from the *in situ* observations to the global climate impact. In practice, this means identifying a series of key processes (as defined by Objectives 1–3), defining a novel observational strategy and innovative framework to look at historical observations with fresh eyes to investigate these processes, pushing the model limits by improving the representation of such processes in numerical models, investigating the extent to which the processes impact large-scale circulation by using numerical frameworks and determining how variability in the processes feedback onto the climate system as a whole (Objective 4). SO-CHIC aims also at contributing to the development of standardized and harmonized marine data (Objective 5). SO-CHIC identified five key scientific questions that need to be tackled to reach the specific objectives listed above. We present these questions in the next section.

## 3. Advances in our understanding of Southern Ocean carbon and heat impact on climate

SO-CHIC has already provided significant advances in our understanding of Southern Ocean carbon and heat impact on climate. In this section, we present a brief overview attempting to summarize the breadth of results achieved to date around five key questions that we have identified as bottlenecks in our understanding of the Southern Ocean’s role on climate.

### (i) **Q1.** Are fine-scale (1–10 km) and transient (day-weeks) processes of the mixed-layer important in driving upper ocean ventilation?

The key processes allowing for the transfer of heat, carbon and other climate-relevant tracers across the Southern Ocean surface interface remain unclear, and their fundamental consequences are not captured by the most sophisticated climate models. In particular, the impact of fine-scale (1–10 km) and transient (day-weeks) processes on the exchange of mass and tracers between the surface and the deep ocean is not fully understood [36]. All of these exchanges are ultimately channelled by the dynamical processes occurring in the mixed layer. Characteristics of the mixed layer (e.g. its depth and buoyancy) are therefore key parameters to understand vertical exchanges across the base of the mixed layer [26].

The mixed layer depth (MLD) is characterized by vertically uniform temperature or density values above it and is commonly calculated from density or temperature profiles [37], though it is known that substantial horizontal buoyancy gradients can exist within the layer that is key to controlling its dynamics. One common assumption is that the mixed layer is actively mixed, but early observations of turbulence demonstrated the difference between the depth of the actively mixed surface layer (referred to as the MLD; XLD) and the MLD [38]. An important difference between the XLD and the MLD is the different timescales over which mixing occurs in these two layers. New observations of upper-ocean turbulence provide a better understanding of the XLD and the impact of transient and fine-scale mixing events (that modify the XLD without necessarily changing instantaneously the MLD) on the ventilation of the near-surface ocean [39–41]. A new physically based definition of the XLD derived from turbulence measurements has been proposed and is more closely correlated with the main sources of turbulence of the upper ocean (i.e. winds, waves and buoyancy fluxes) than the MLD [40].

**Figure 1.**
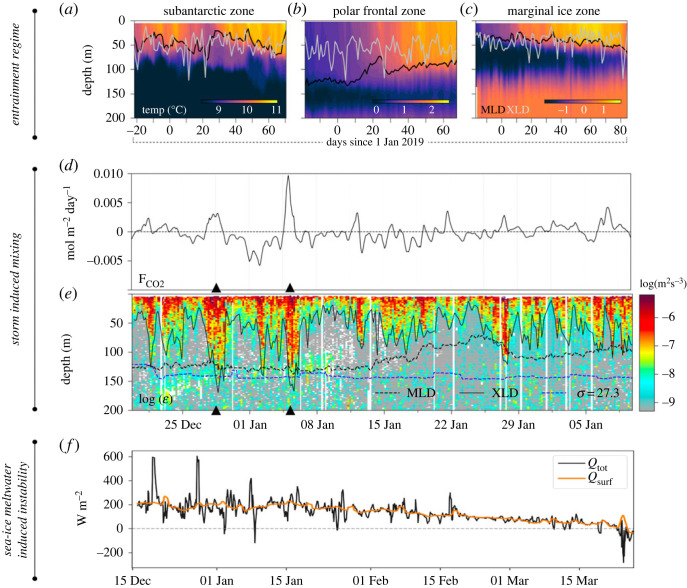
Upper ocean ventilation processes. (*a*–*c*) Vertical sections of temperature (${\;}^{\circ }C$) in early 2019 in the Subantarctic Zone, Polar Frontal Zone and Marginal Ice Zone, respectively. Lines indicate the mixed layer depth (MLD; black) and mixing layer depth (XLD; grey). Note the colourbar limits vary but are scaled to a range of $2.5^{\circ }C$. (*d*–*e*) Observed air–sea ${\mathrm{CO}}_{2}$ flux ($F_{{\mathrm{CO}}_{2}}$; *d*) and underlying vertical section of dissipation rate of turbulent kinetic energy (ϵ; *e*) in early 2019. The solid black line is the mixing-layer depth (XLD), the dashed black line is the mixed layer depth (MLD) and the blue dashed line is the $27.3\;{\mathrm{kg}\;m}^{-3}$ isopycnal indicating the upper bound of upper circumpolar deep water. (*f*) Sum of the total surface buoyancy forcing, restratification due to mixed layer eddies and wind-driven submesoscale Ekman transport expressed as an equivalent heat flux (*Q*${\;}_{\mathrm{tot}}$; black), and total surface buoyancy forcing alone ($Q_{\mathrm{surf}}$; orange) in early 2018. Panels (*a*–*c*) are adapted from du Plessis *et al.* (2022) [42]; (*b*) from Nicholson *et al.* (2022) [43]; (*c*) from Giddy *et al.* (2021) [44]. (Online version in colour.)

Knowledge of the XLD was instrumental in delineating different entrainment regimes across the Southern Ocean (figure 1*a*–*c*; [42]). In particular, it allowed to pinpoint the role of summertime buoyancy forcing and wind-driven processes on the intraseasonal (1–10 days) mixed layer thermohaline variability in three Southern Ocean regions important for water mass transformation: the Subantarctic Zone, the Polar Frontal Zone and the Marginal Ice Zone. In the Subantarctic Zone and the Marginal Ice Zone, shallow MLD and strong stratification enhanced mixed layer buoyancy gain by trapping incoming heat, while frequent transient mixing events, evidenced by the XLD, resulted in buoyancy loss due to the entrainment of cold, salty water from below (figure 1*a*,*c*; [42]). By contrast, in the Polar Frontal Zone rapid mixing, linked to Southern Ocean storms, set a persistently deep MLD and reduced the transient entrainment of cold water from below because the XLD lies mostly within the MLD (figure 1*b*; [42]).

Deep mixing events that surpass the depth of the MLD can have large consequences and have been shown to potentially exert major influences on our understanding of the role of the Southern Ocean in climate [43]. For instance, deep mixing events caused by large synoptic storm-driven ocean variability have been observed to be able to deepen the XLD below the MLD, entraining carbon-rich deep waters lying below the MLD. Such entrainment of carbon into the mixed layer shapes the air–sea gradient of ${\mathrm{CO}}_{2}$, and ultimately the potential outgassing of carbon from the Southern Ocean to the atmosphere (figure 1*d*,*e*; [43]). While an unprecedented set of concurrent autonomous observational platforms was able to fully capture such events (figure 1*b*), extrapolation across the subpolar Southern Ocean demonstrated the importance of those fine-scale and transient events on the functioning of the Southern Ocean carbon budget [43]. Further south, in the Marginal Ice Zone, sea–ice mostly shields the upper ocean from storm events, but bursts of meltwater released from sea–ice have large implications at small scales (meso- and submesoscale). Such meltwater events create fronts of importance to Southern Ocean water-mass transformation [44]. Sea–ice meltwater strongly controls the buoyancy of the mixed layer during early summer, with large transient (1–10 days) buoyancy fluxes (figure 1*f*; [44]), and the generated mesoscale meltwater lateral gradients allow for the growth of mixed layer eddies confined to the surface boundary layer.

In summary, the unprecedented combination of new *in situ* observations ranging from turbulence measurements to observations of the air–sea gradient of ${\mathrm{CO}}_{2}$, through measurements of the thermohaline properties of the upper ocean, demonstrates the importance of fine-scale (1–10 km) and transient (days to weeks) processes of the mixed layer for local ventilation of the Southern Ocean. Work is underway to better capture the net effect of such small-scale processes on the large-scale Southern Ocean circulation and impact on climate.

### (ii) **Q2.** What are the drivers of the large-scale horizontal circulation in the subpolar Southern Ocean and how does this circulation relate to large polynya events?

Despite its central role in the global climate, the subpolar Southern Ocean circulation is arguably still one of the least understood ocean circulation systems of the planet. We know that it is characterized by large-scale regional gyres in the Weddell and Ross Seas, which are bounded at their northern edge by the eastward-flowing Antarctic circumpolar current (ACC), and at their southern edge by the westward-flowing Antarctic slope current (ASC). But our understanding of the dynamical drivers of this circulation system is challenged by the lack of large-scale observations of the circulation in the seasonally sea–ice-covered sector of the Southern Ocean.

**Figure 2.**
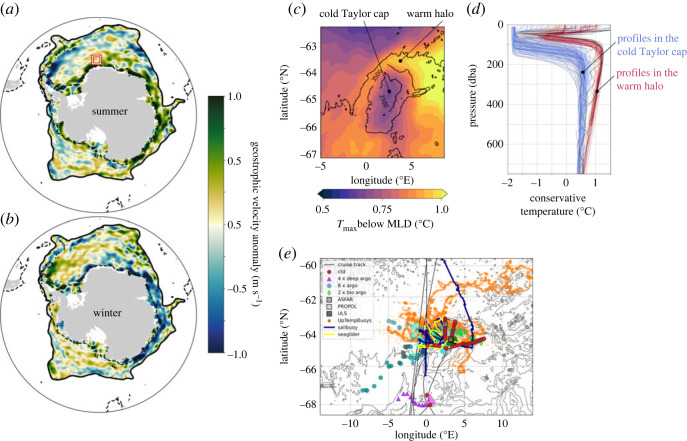
Large-scale ocean circulation and drivers of deep convection. Zonal surface current anomalies (relative to the annual mean) of the subpolar Southern Ocean in (*a*) Summer (December, January, February; DJF) and (*b*) winter (June, July, August; JJA) from a novel processing of satellite altimetry in sea–ice-covered regions [45,46]. The dotted line is the 1000 m isobath. The red rectangle shows the region of Maud Rise highlighted in *c*. (*c*) 2010–2019 mean of the temperature maximum below the mixed layer depth from the GLORYS12 reanalysis [47] in the Maud Rise area. Superimposed black contours represent the bottom topography. The horizontal circulation creates a relatively warm water halo circulating around a cold Taylor cap. (*d*) Vertical profiles of conservative temperature as observed in the Maud Rise region by profiling floats. All profiles are coloured according to their sampling location. Blue indicates profiles sampled in the cold Taylor cap (where the bathymetry is <3500 m); red indicates profiles sampled in the warm water halo (bathymetry >3500 m). Profiles with evidence of water mass intrusions (by visual inspection) are shown in black. (*e*) New set of observations acquired in January 2022 in the Maud Rise area. The figure shows the locations of observations from ship CTD (red circle), different type of profiling floats (purple triangle, blue circle, green diamond), mooring lines (grey squares), surface buoys (orange circle), surface autonomous vehicle (blue line) and profiling glider (yellow line). Panels (*a*,*b*) are adapted from Auger *et al.* (2022) [46] and (*d*) from Mohrmann *et al.* (2022) [48]. (Online version in colour.)

This major constraint has been partially alleviated by a new sea level anomaly (SLA) product of the seasonally sea ice-covered Southern Ocean, which combines observations from multiple satellites, and state-of-the-art sea–ice capable processing [45]. These observations reveal the seasonal cycle of the geostrophic circulation in the subpolar Southern Ocean (figure 2*a*,*b*) [46]. It is primarily explained by three main modes of variability: a first mode corresponding to a winter acceleration of the Weddell and Ross gyres, consistent with large-scale variability of wind stress curl, through Sverdrup dynamics; a second mode associated with a winter intensification of the ASC, forced by easterly wind variability on the continental shelf, with a circumpolarly propagating signal consistent with the so-called Southern Mode; and a third mode corresponding to a mid-season northward progression of the ASC acceleration/deceleration that is consistent with a local response to surface stress modulated by the combined seasonal cycles of sea ice and wind stress.

This horizontal circulation system is intertwined with important water-mass transformation, especially in regions where it interacts with bottom topography. In particular, new observations have highlighted the importance of the horizontal circulation system around Maud Rise in creating favourable conditions for the formation of Maud Rise polynyas [48]. The horizontal circulation wraps a halo of relatively warm water around a colder Taylor Cap that sits on top of Maud Rise (figure 2*b*) [49]. This allows efficient stirring and mixing across the distinct water-masses, as evidenced by high-frequency profiling floats that have highlighted temperature-salinity intrusions indicative of enhanced lateral and vertical mixing along heavily sloping isopycnals (figure 2*d*) [48]. The combination of SO-CHIC *in situ* observations (figure 2*e*) with other historical datasets has also revealed the large interannual variability in warm water properties in the Halo and the Taylor Cap, remotely forced by advection of Weddell Gyre deep waters in the region [50]. A warming of deep waters is observed in the Taylor Cap during the years preceding the opening of the Polynya in 2016 and 2017, starting in 2011. Anomalies in the Ekman transport of salinity across a jet girdling the northern flank of the Maud Rise, driven by intensified down-front surface stresses during 2015–2018, helped weaken the stratification in the region [51]. Ultimately, this interplay between the large-scale current system and local mixing preconditions the formation of an open-ocean polynya and the ventilation of the deep ocean in this key location of the Southern Ocean.

In summary, novel processing of past satellite observations combined with state-of-the-art *in situ* observations from autonomous profiling floats and high-resolution ocean modelling allows to open a new door in our understanding of the drivers of the large-scale circulation and associated water-mass transformation in the subpolar Southern Ocean. At large-scale, wind-stress is a major driver of the seasonality of the subpolar ocean circulation, both for the system of gyres, and for the continental slope current. At Maud Rise, the horizontal currents combined to local mixing processes are able to precondition the formation of open ocean polynyas.

### (iii) **Q3.** What are the processes controlling the origin and the fate of bottom waters globally?

Carbon and heat can enter the Southern Ocean through the formation, export and consumption of bottom waters. The Weddell Gyre is one of the key regions for Antarctic Bottom Water (AABW) formation and ventilates much of the Atlantic Ocean [52]. The AABW has been observed to be warming, which contributes to sea-level rise [2]. However, the drivers of such change and variability are difficult to determine due to the poorly understood and observed dynamics of dense water formation and export within the Weddell Sea, and due to their unrealistic representation in climate models [53].

**Figure 3.**
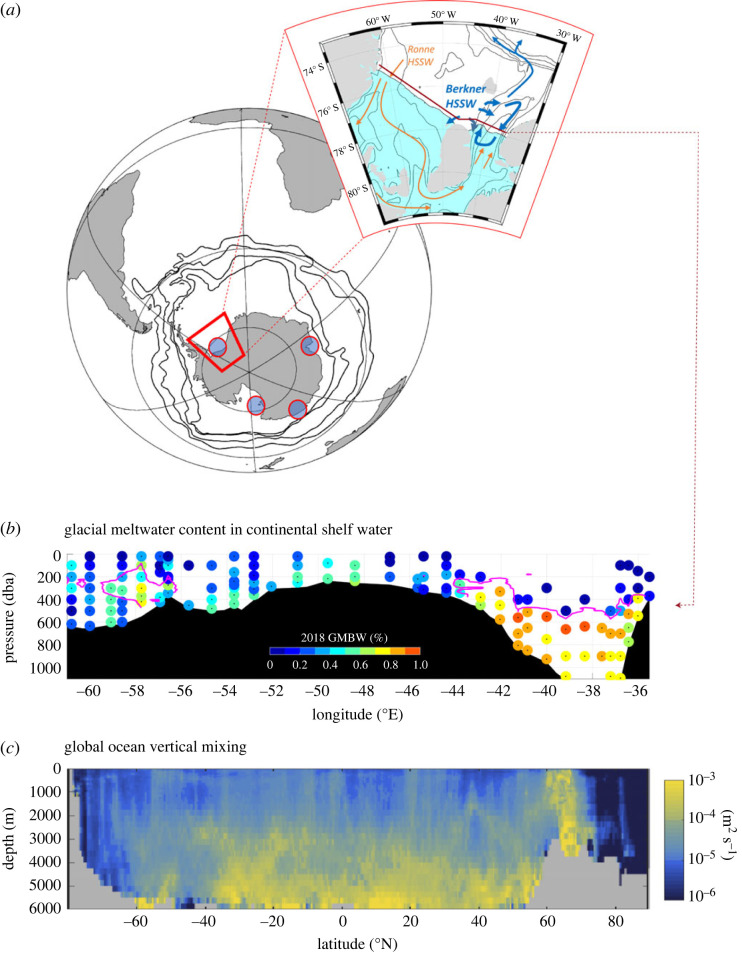
The origin and fate of bottom waters. (*a*) Map showing the four main Antarctic Bottom Water formation sites around Antarctica (blue circles). The black contours show the position of the four main fronts of the Antarctic Circumpolar Current. The inset shows a zoom on the southern Weddell Sea Region, with a schematic of the regional circulation of dense waters (Ronne and Berkner HSSW) on the continental shelf (blue arrows) and within the Filchner–Ronne Ice-Shelf cavity (orange arrows). The sub-ice-shelf cavity is shaded in cyan. The red line directly to the north of the cavity indicates the location of the vertical section shown in *b*. (*b*) Glacial basal melt water fractions (%) along the ice shelf edge shown by coloured dots at the sample location and depth in 2018. Magenta contours mark the $-2.0^{\circ }C$-isotherm as an indicator for dense shelf water outflow. (*c*) Zonal mean diapycnal diffusivity of the world ocean due to tides. Diffusivity averages have been weighted by the local stratification. The inset in (*a*), and (*b*) are adapted from Janout *et al.* (2021) [54]. Panel (*c*) is adapted from de Lavergne *et al.* (2020) [55]. (Online version in colour.)

The ventilation of the deep ocean by bottom water can be conceptualized as a two-step process. First, interactions between the atmosphere, ocean and cryosphere lead to the formation of Dense Shelf Water that eventually overflows the shelf break and entrains ambient waters on its way to the bottom of the ocean. Second, bottom water and the tracers it contains slowly mix with the overlying layer. New observations at the southern edge of the Weddell basin have allowed us to revisit the circulation and water-mass transformations that lead to dense water formation in the Weddell Sea (figure 3*a*,*b*) [54]. Observations of noble gases taken at the outer boundary of the Filchner–Ronne sub-ice-shelf cavity allowed to estimate the fractions of glacial meltwater contained in water masses as well as the circulation timescale within the cavity. The cavity is mostly filled via the western end of the section, where a low glacial meltwater fraction is observed, and the dense shelf water outflows from the cavity on its easternmost side (Filchner Trough), associated with relatively high glacial meltwater content (0.8–1%) (figure 3*b*). The total travel time within the cavity is estimated to be around 6 years [54]. This unique set of observations highlights the importance of the interactions between dense shelf water and the ice-shelf in setting the properties of the dense water before it overflows the sill of the continental shelf (figure 3*b*).

While a fraction of the overflown dense waters cascades downslope and fills the bottom of the Weddell Sea, another fraction of these dense waters flows along the continental slope steered by bathymetry [56,57], and part of it is eventually exported to the global ocean in the form of AABW through deep passages located in the northern Weddell Sea [27]. Variability of the dense water plume (velocity and location) flowing along the continental slope is associated with a wide range of timescale. Interannual variability is associated with climate modes (Southern Annular Mode; El Nino Southern Oscillation), and it superimposes on lower frequency change, as observed from a set of mooring at the tip of the Antarctic Peninsula [58]. Low-frequency change also shows up in historical repeated observations. Ongoing work [59] shows how the densest layer of the Weddell Sea has been shrinking over the past decades, being slowly replaced by lighter water-masses. This multi-decadal reduction of bottom water volume is linked to a decrease of dense shelf water production on the southernmost continental shelf of the Weddell Sea, which reduces the export of dense water down the continental slope, because of change in sea–ice production [59].

When leaving the ice shelf cavity northward, the dynamics of the overflow of dense shelf water is strongly connected to southward transport of the relatively warm water, which triggers more basal melt in the ice cavity, as observed by a set of mooring placed at the continental shelf sill next to the Filchner Trough [60]. Ultimately, the northward-flowing (downslope) deep-water plumes form bottom water reservoirs, which ventilate the deep ocean via bottom ocean mixing, diffusing tracers throughout the deep ocean [27]. As such, it is essential to correctly represent bottom water mixing in numerical models, but existing parameterizations remain one of the major challenges for ocean models [53]. This issue should be tackled by advances in process understanding in tandem with refinement of existing parameterization schemes. For instance, an energy-conserving mixing scheme was proposed based on a state-of-the-art physical understanding of internal tides, accounting for the local breaking of high-mode internal tides and the distant dissipation of low-mode internal tides (figure 3*c*) [55]. Such new advances, which are consistent with available microstructure observations and with upper-ocean finestructure mixing estimates [55], enable step changes in our understanding of deep-ocean ventilation. Bottom-generated mixing is more confined to the deep ocean than previously thought (figure 3*c*), implying notably that the overturning of bottom water in the Pacific is confined beneath a mid-depth Pacific shadow zone shielded from mean advection [61]. This novel view based on refined understanding of bottom ocean processes is a radical departure from the previously prevailing paradigm proposing that Pacific bottom water would upwell to depths as shallow as 1.5 km.

In summary, important improvements in our understanding of bottom water formation and consumption were based on novel observations and theoretical advances. In the Weddell Sea, processes within the sub-ice-shelf cavity and over the continental shelf are key for setting properties of bottom waters and controlling such waters’ outflow as they sink down the continental slope to the abyssal ocean. Upon reaching the ocean floor, the geographical and depth pattern of tidal mixing is key to shaping the global overturning of bottom water. Refined understanding of deep-ocean mixing radically changed our view of the global ocean overturning. In addition, the refined process understanding of the origin and fate of world ocean bottom waters is currently being used to shed light on the causality of observed multi-decadal change of bottom ocean properties in the Southern Ocean.

### (iv) **Q4.** How is the thermohaline structure of the Southern Ocean responding to climate change?

Upper ocean processes (Q1), polynya events (Q2) and bottom water formation and consumption (Q3) combine to ventilate the Southern Ocean. This ventilation allows to propagate climate change signals at depth, which modify the Southern Ocean thermohaline structure. These changes are unequivocal when looking at multi-decadal repeated observations of the Southern Ocean.

**Figure 4.**
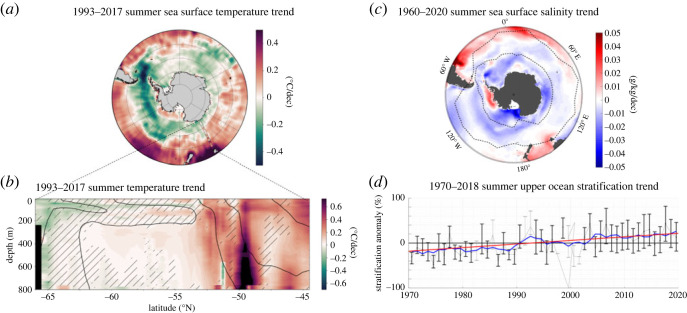
Observed long-term change of the Southern Ocean. (*a*) Summer SST Trends (${\;}^{\circ }C/\mathrm{dec}$) from 1993 to 2017 (November–February). Black line indicates the region of the SURVOSTRAL transects shown in *b*. (*b*) *In situ* temperature trends (${\;}^{\circ }$C/dec) from SURVOSTRAL XBT observations. Hatched regions represent zones where abs(Trends∗ΔT)/STD<1, ΔT being the length of the record, and STD the standard deviations of annual departure from the trend; i.e. where the trends are smaller than the interannual variability over the 25 years of measurements. (*c*) Ocean sea surface salinity trends (g/kg/dec) for 1960–2017 from a reconstruction based on historical observations. (*d*) Long-term (1970–2018) change in upper ocean summer stratification (% of the climatological mean) in the domain shown by the black contours in *c*. The panel shows: the annual median percentage anomaly (thin grey line; from the local climatological seasonal cycle), computed from individual historical observation; error bars referring to the 33th–66th percentile range of percentage anomaly (error bars are shown in black (grey) when more (fewer) than 50 data points are used in the annual statistics); the associated 5-year smoothed median time series superimposed in blue; and a linear trend in 1970–2018, shown by the red line if greater than twice its standard error. Panels (*a*,*b*) are adapted from Auger *et al.* (2021) [62], (*c*) from Cheng *et al.* (2020) [63] and (*d*) from Sallée *et al.* (2021) [64]. (Online version in colour.)

For upper-ocean temperature, the longest time-series resolving some of the seasonal cycle is a 25-year time series of the upper 800 m repeated several times a year across the Southern Ocean between Tasmania and Antarctica [62]. This repeated section provides a unique vertical view of multi-decadal temperature change of the Southern Ocean allowing to complement the sea surface temperature change provided by satellite observations (figure 4*a*,*b*). Three regions stand out as showing large temperature changes that dominate over interannual variability: on the northern part of the section (north of $52.5^{\circ }\;S$), subantarctic waters have warmed at a rate of 0.29±0.09${\;}^{\circ }$C per decade from 1993 to 2017; by contrast, on the southern part of the section (south of $60^{\circ }\;S$), near-surface subpolar waters have cooled at a rate of $-0.07\pm 0.04^{\circ }C$ per decade; in between these two sectors ($60^{\circ }S\text{--}55^{\circ }\;S$) and at depth, the subsurface subpolar deep waters have warmed at a rate of $0.04\pm 0.01^{\circ }C$ per decade [62].

The warming of the subsurface subpolar deep waters is the most robust long-term trend observed in the Southern Ocean when compared with its weak interannual variability. Also observed in subpolar seas is a large shoaling of the subsurface temperature maximum, at a rate of 39±09 m per decade [62]. Such warming and shoaling of the Southern Ocean deep water is one of the key drivers of Antarctic ice sheet mass loss [2], which releases freshwater at the Southern Ocean surface, and contributes to its freshening [2]. Overall, ice sheet mass loss, water cycle amplification and sea ice regime changes conspire to sustain a multi-decadal decrease of surface salinities across much of the high-latitude Southern Ocean. The relative contribution of each factor remains poorly quantified, despite regional constraints obtained with analysis of water stable isotopes [65]. The freshening of the Southern Ocean is clear when considering long-term change of sea surface salinity since 1960 (figure 4*c*) [63]. Globally, it contributes to an amplification of the sea surface salinity pattern (low salinity regions become less salty, and high salinity regions become more salty), from which one can derive an estimate of the global atmospheric water cycle amplification of 2.6%±4.4%/K since 1960 [63].

Decreased sea surface salinity in the Southern Ocean has direct consequences for ventilation since it drives an increase of the upper-ocean stratification (figure 4*d*) [64]. By recovering oceanographic observations globally from 1970 to 2018, we showed that the upper ocean stratification at the base of the summer mixed layer has increased by 8.9±2.7% per decade globally ($10^{-5}\text{--}10^{-4}$ per second squared per decade, depending on region), with a very marked change in the Southern Ocean (figure 4*d*). Whereas prior work has suggested that a thinner mixed layer should accompany a more stratified upper ocean, we find instead that the summertime mixed layer in the Southern Ocean has deepened by several metres per decade (typically 5–10 m per decade), possibly due to increased wind-induced turbulence [64]. However, the potential change in processes that can lead to increased turbulence remains poorly understood (see Q1).

In summary, the Southern Ocean has experienced vast changes of its thermohaline characteristics over the past decades. Despite suffering from a significant lack of historical observations due to its remoteness and harsh climatic conditions, past observational efforts have been used to demonstrate significant changes both at the surface and at depth. Large warming of the northern edge of the Southern Ocean is a fingerprint of the key role that the Southern Ocean plays to extract heat from the atmosphere and store it at depth. Further south, the gentler warming observed at depth is a clear departure from interannual variability and can have dramatic consequences for the melt of Antarctic ice shelves. Following changes in sea ice, continental ice and precipitation, the surface of the Southern Ocean freshens, which increases its stability, with potentially large consequences for the future of the Southern Ocean overturning circulation. The impacts of these changes on global climate through change in overturning rate, change of pattern effect, or through a potential positive feedback loop between warming and ice shelf melt, remain an active area of research.

### (v) **Q5.** What are the critical sensitivities and processes that control large-scale Southern Ocean change under climate change?

The surface climate of planet Earth is determined by a complex set of interactions among the atmosphere, the ocean, the cryosphere and the land. In the Southern Ocean, these interactions involve physical, chemical and biological processes, many of which are only understood to some exent, which blurs our understanding of the critical sensitivities of the Southern Ocean climate system.

For instance, we showed that the continued meltwater discharge of the Antarctic ice sheet and ice shelf of the Southern Ocean can have global implications with, e.g. regional warming in East Asia via atmospheric teleconnections [66]. Atmospheric patterns and climate modes can in turn have important implications for the melt of Antarctic ice shelves [67], for the thermohaline properties of the dense water plumes both at source regions [54], during their export downslope [58], and in consequence for bottom water properties [59]. The use of an unprecedented set of observations from hot-water drilled boreholes in the Filchner–Ronne ice shelf allowed to show the importance of the Amundsen Sea Low position in setting the cavity circulation and its melt rate. The analysis suggests a predominantly wind-driven control on sea ice formation at the edge of the cavity, which has a direct impact on the cavity-wide buoyancy-driven circulation and the associated dense water outflow [67].

**Figure 5.**
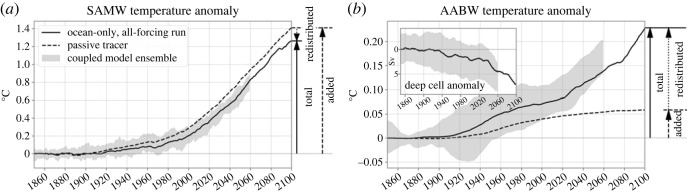
Processes driving Southern Ocean change. Temperature anomalies in (*a*) Subantarctic Mode Water (SAMW) and (*b*) Antarctic Bottom Water (AABW) as projected by the IPSL climate model (IPSL-CM6A-LR; the grey shading represents the envelope of the large ensemble until 2059) under a moderate future emission scenario (SSP2-4.5). Anomalies are expressed relative to 1850–1899. The plain line represents the total temperature change in the ocean-only framework mimicking the coupled model historical+SSP2-4.5 experiment, relative to a CTL simulation to remove the maximum effects of internal variability at each time step. The dashed line shows the temperature change associated with surface heat flux anomalies that would enter the ocean and be passively propagated by the ocean circulation (added heat). The difference between the total temperature anomaly and the added temperature anomaly represents the temperature anomaly caused by changes in the circulation caused by climate change (redistributed heat). The inset in (*b*) is the time series of the anomaly of the strength of the Southern Ocean deep cell (minimum of the global meridional streamfunction between $30^{\circ }\;S$ and $80^{\circ }\;S$ below 2000 m). Adapted from Silvy *et al.* (2022) [30].

At larger scale, variability and change of wind-stress patterns can have major implications for Southern Ocean water-mass characteristics, in combination with changes in heat and freshwater air–sea fluxes. We developed a new modelling framework to disentangle the processes leading to ocean temperature and salinity change in a large ensemble of simulations of a global climate model [68]. When focusing on upper-ocean water masses of the Southern Ocean (figure 5*a*), the anthropogenic warming is almost exclusively explained by the uptake of heat at the ocean surface that is then passively exported by the ocean circulation (added heat in figure 5*a*) [30]. In comparison with changes in air–sea heat fluxes, circulation changes have a relatively minor role (redistributed heat in figure 5*a*). On the contrary, in the deeper ocean, the slowdown of the deep overturning circulation (figure 5*b*, inset) responding to increased near surface stratification plays a large role in setting the timescales and amplitude of ocean warming (figure 5*b*) [30]. We see here how different water masses can respond differently to human-induced atmospheric changes. These water-mass specific changes are associated with water-mass specific feedbacks on the carbon cycle [69]. Indeed, as water masses change due to climate change they either enhance or reduce their ability to take up carbon from the atmosphere. Under increasing greenhouse gas emissions, all Southern Ocean water masses increase their ${\mathrm{CO}}_{2}$ uptake due to continued increasing atmospheric concentration [69]. But we showed that this increase is offset by about 25% due to feedbacks associated with water-mass characteristics and circulation changes that tend to decrease ${\mathrm{CO}}_{2}$ uptake, particularly in Subantarctic Mode Waters [69]. However, in the coming century, the Southern Ocean is projected to continue to be a major sink for both heat and carbon [4]. The processes behind the interplay of heat and carbon uptake in the Southern Ocean have an important influence in determining the transient climate response to cumulative ${\mathrm{CO}}_{2}$ emissions (TCRE), and the warming commitment after cessation of greenhouse gas emissions [4].

In summary, Southern Ocean changes are strongly connected with the global climate. While atmospheric teleconnections can drive regional atmospheric warming in Asia in response to changes in the melt of Antarctic ice, variability in large-scale atmospheric circulation patterns has been observed to directly control local ocean circulation beneath ice shelves in the Weddell Sea. More generally, changes in large-scale atmospheric circulation combined with changes in air–sea heat and freshwater fluxes impact Southern Ocean water-mass characteristics. Bottom water-mass characteristics are more sensitive to ventilation changes mediated by change in the stratification, and upper-ocean water-mass characteristics are more sensitive to changes in buoyancy flux propagating in the interior by the mean circulation. These water-mass specific changes have distinct effects on the ${\mathrm{CO}}_{2}$ uptake of the Southern Ocean. Ultimately, the interplay between processes driving heat and carbon change in the Southern Ocean interior have an important impact on the response of global climate to greenhouse gas emissions.

## 4. Conclusion

The SO-CHIC programme was launched with the aim to improve our understanding of the Southern Ocean’s role in climate. The present paper presents the programme and its overall objectives, and takes stock of the progress halfway to its completion.

Five main questions have been identified as inhibiting progress in our understanding of heat and carbon uptake by the Southern Ocean and its impact on climate. Three of them target key aspects of Southern Ocean processes that control ocean ventilation: small scale (1–10 km) and transient (day-weeks) processes of the upper ocean; drivers of the large-scale circulation system and their impact on water-mass transformation; local processes at the origin of bottom water production on the Antarctic continental shelf, and mixing processes consuming these bottom waters in the abyss. Two questions focus on past and future changes of the Southern Ocean and potential feedbacks, and on the critical sensitivities of the system that could change its role in mitigating climate change in the future.

We showed that local and transient processes such as storm-induced turbulence, or mesoscale and submesoscale processes developing in the open ocean or at the sea–ice edge, might be instrumental in the transfer of tracers across the base of the mixed layer. Regionally, around Maud Rise, remote advection of anomalous water masses and cross-frontal flux have the ability to create favourable conditions for the formation of an open ocean polynya that provides a direct connection between the surface ocean and the deep interior. Such cross-frontal flux is particularly efficient because the large-scale horizontal circulation creates a sharp water-mass front that is maintained around the edge of Maud Rise. The strength of the large-scale circulation itself evolves seasonally and this seasonality is strongly controlled by the winds and slightly influenced by sea–ice coverage. Sea–ice is however key in forming dense water on the Antarctic continental shelf, and this dense water is then transformed by its interaction with Antarctic ice shelves and by entraining warmer ambient waters when cascading downslope. The export of dense shelf waters off the Weddell continental shelf occurs mainly along canyons and is dynamically connected with the import of relatively warm deep water onto the continental shelf. Ultimately, the bottom waters produced in the Weddell Sea, including the fraction of them escaping the Weddell Sea, are consumed by abyssal mixing processes, which control the shape of the deep overturning circulation. New understanding of deep mixing intensity shows that large regions of the ocean are disconnected from the global water-mass overturning.

This set of processes shapes the ventilation of the Southern Ocean, which allows for a deep propagation of the climate change signal. The Southern Ocean has changed rapidly in the past three to six decades. Large and deep warming has overcome interannual variability in many water masses. This is accompanied by a freshening of its surface waters, which is explained by a combination of ice shelf melt, change in sea ice regime and increasing precipitation rates. This surface salinity change has important consequences for the stratification, which is increasing at a pace much larger than previously thought. Climate models and theoretical understanding suggest that such an increase in stratification reduces the strength of the deep overturning, which reduces the influx of cold water from the surface ocean, and therefore warms the abyssal Southern Ocean. By contrast, water masses resulting from the ventilation of the upper 1000 m, north of the ACC warm not because of a change in circulation but mostly because of the passive propagation of heat gained at the surface. Delineating these processes and how carbon content change might differ from heat content change is fundamental to our understanding of future global climate, because this carbon-heat nexus provides a first-order influence in determining the transient climate response to cumulative ${\mathrm{CO}}_{2}$ emissions, which is at the forefront of current international climate negotiations.

The SO-CHIC programme continues. The foundation that has been built in the first part of the project is now efficiently delivering results to further improve our understanding of the Southern Ocean and its role in global climate.

## Data Availability

The data are provided in the electronic supplementary material [70].
